# Microvasculature evaluation of anisometropic amblyopia children by Angio-OCT

**DOI:** 10.1038/s41598-023-29816-1

**Published:** 2023-02-16

**Authors:** Haiyun Ye, Siying Wang, Yidan Zhang, Wangyi Fang, Han Ye, Luya Chen, Tong Qiao

**Affiliations:** Department of Ophthalmology, Shanghai Children’s Hospital, School of Medicine, Shanghai Jiao Tong University, No.355 Luding Road, Shanghai, 200062 China

**Keywords:** Paediatric research, Vision disorders

## Abstract

To compare and assess the choroidal and retinal microstructural vascularity in amblyopic eyes with the fellow eyes in anisometropic amblyopic children using angiography optical coherence tomography (Angio-OCT). Twenty-seven children (54 eyes; 5.59 ± 1.08 years old; 59.3% girls) were enrolled in this study. Choroidal thickness (CT) was measured with the use of the enhanced depth imaging mode in Angio-OCT. Parafoveal/peripapillary vascular density indices and the foveal avascular zone (FAZ) size were analyzed by MATLAB code programming on Angio-OCT images. The mean FAZ size of the amblyopic eyes were larger both in superficial and deep capillary plexus layer (SCPL/DCPL). Compared with the contralateral eyes (BCVA were normal), all the vascular density indices of SCPL and DCPL in the parafoveal and peripapillary zones were lower in the amblyopic eyes, however, the difference was insignificant (*p* > 0.05). No significant decrease was observed in four quadrants analyses of the amblyopic eyes (*p* > 0.05). Except for the measurement at 2000 µm and 1500 µm from the fovea in temple, CT in amblyopic eyes were significantly thicken than the fellow eyes (*p* < 0.05). Compared with the fellow eyes, the CT of certain areas were thicker in the amblyopic eyes. Though the FAZ size of the amblyopic eyes was larger in SCPL/DCPL layers, the retinal vascular density indices in SCPL/DCPL were lower in amblyopia eyes without statistical difference. Angio-OCT may be an effective way to evaluate the status of the choroidal and retinal vascular system in amblyopic children.

## Introduction

As a common cause of preventable vision loss in children, amblyopia may be defined as a loss of visual acuity in one or both eyes due to abnormal visual processing. This may occur during the critical period of visual development without anatomical changes and the best corrected visual acuity (BCVA) did not develop to the normal level in one or both eyes^1,2^. Unilateral amblyopia may be caused by anisometropia, strabismus or visual deprivation. This may be clinically defined as a difference of 0.20 logMAR (Logarithm of the mean angle of resolution) or more in BCVA between the two eyes^3^. For example, interocular differences of > 1 diopters of refractive error is an amblyogenic factor for anisometropic amblyopia^1,2^. Though the pathophysiology of anisometropic amblyopia have not been fully understood, the functional variation involved in its development can occur at different levels of the visual pathway^4^. Many researchers found structural and functional deficits changes occur in the primary and extrastriate visual cerebral cortex areas as well as the choroid and retina in the eyes^1,2,5–8^. However, Araki et al. found that, though the amblyopic eyes had smaller FAZ area of SCP , there was no significant difference in the macular vessel density between the amblyopic and fellow eyes^9^. Assessing the foveal avascular zone and vessel density of retina, Demirayak Bengi and colleagues found no difference between amblyopic eyes, controls, and fellow eyes of patients with unilateral amblyopia^10^. These results about the microstructural vascularity were either inconsistent or contradictory.

Without dye injection, angiography optical coherence tomography (Angio-OCT) is noninvasive and quantifiable in microvascular vessel perfusion of fundus^11–13^. This quantitative assessment technique could sensitively detect the retinal vessel minor alteration. We aim to compare the amblyopic eyes with the fellow eyes in anisometropic amblyopic (defined by the BCVA in one eye was 0.20 logMAR lower or by more than the age standard while the BCVA in the contralateral eye reached the age standard) children and assess the microstructural vascular differences by Angio-OCT.

## Materials and methods

### Amblyopia patients

Pediatric patients who were diagnosed unilateral anisometropic amblyopia were examined by Angio-OCT from April 2019 to July 2020 in Shanghai Children’s Hospital. Full ophthalmologic examinations, including refraction (cycloplegic refraction), visual acuity testing, slit-lamp biomicroscopy and dilated fundus examination, were preceded in all participants. LogMAR units was used to measure BCVA for statistical analyses. Taking the differences in children's cognitive abilities and cooperation into account, we converted Snellen into logMAR equivalents in the present study. Inclusion criteria of the enrolled patients were following: age between 4 and 7 years old, anisometropic amblyopia, without ocular or systemic diseases. Exclusion criteria were as follows: binocular amblyopia, with any ocular disease history or any systemic disease, previous ocular surgery or laser therapy history, use of any systemic medicine in recent 6 months, with treatment for amblyopia, and with esotropia or exotropia of ≥ 10Δ.

### Retinal and choroidal data

All ocular measurements were performed at the same period in the morning (between 9:00 a.m. and 11:00 a.m.). The definition of choroidal thickness (CT) was the vertical distance from the retinal pigment epithelium (RPE) outer surface to the scleral inner surface^14–16^. The choroid was measured from subfoveal area to 2000 µm both at the nasal (N2000) and the temporal (T2000) with 500 µm intervals by the use of the enhanced depth imaging (EDI) mode in Angio-OCT^14^. An annulus area outside foveal avascular zone (FAZ), which was the central area devoid of vessels, was set as parafoveal area, which the outer diameter was 3-mm and a 1-mm of the inner diameter^17^. The peripapillary area was set as a 700-µm-wide elliptical annulus extending outward from the optic disc demarcation^18,19^. The vascular density was defined as the occupied vessel lumens percentage in the corresponding segmented areas^19^.

### Angio-OCT

To avoid the related risks of indocyanine green and fundus fluorescein angiography, Angio-OCT had been applied frequently in many reports of children with its noninvasive advantage. The Angio-OCT images on the parafoveal/peripapillary areas of the observed eyes were captured by Zeiss Cirrus 5000-HD-OCT with pattern 6 mm × 6 mm. Quantitative mensuration of the macular and optic nerve head vessel density indices containing vessel diameter (VD), vascular skeleton/area density (VSD/VAD), index of vessel perimeter (IVP) on the superficial/deep capillary plexus layer (SCPL/DCPL), and the FAZ size were assessed and compared. The boundary from 3 mm beneath the internal limiting membrane to 15 mm beneath the inner plexiform layer to 70 mm beneath the inner plexiform layer were defined as the SCPL and the DCPL separately^17^. These indicators were employed to reflect the vascular perfusion not only from the two dimensions but also from the three-dimensional perspective. According to the quantification of Angio-OCT images, we compared the vascular density of retinal network in unilateral anisometropic amblyopia children of the two eyes in our study.

### Data treatment and statistical analysis

The data analysis algorithm was coded by MATLAB (2021) code programming, which objectively analyzed the acquired data from the digital images of Angio-OCT. It used the algorithm based on the optical microangiography (OMAG), which was a method for obtaining three-dimensional images of blood vessels within a volume of tissue. A black and white binary image was obtained by segmenting the vessels in the OMAG image. Using a low-pass filter, a global threshold was set, and a local adaptive threshold was implemented to binarize the image based on the average pixel value within a predefined window size. The algorithm of the vascular indices was previously described in detail^20^. All values were represented in the mean ± standard deviation (SD) form. SPSS (Version 23; Chicago, IL, USA) were applied for statistical analyses, and *p* < 0.05 was defined as statistical significantly difference. The paired sample t test was applied to compare the differences between amblyopic eyes and fellow eyes. To ensure the reproducibility of measurements, all subjects were examined three times or more by the same technician. Choroidal parameters were measured three times to get the average of the measurements, while the Angio-OCT retinal vascular scans were performed three times or more and the images with an image quality value (range 0–10) higher than 8 were selected.

### Statement of ethics

The research protocol adhered to the tenets of the Declaration of Helsinki and was approved by the Institutional Review Board of the Shanghai Children’s Hospital (2018R013-E02). Written informed consent was obtained from the participants' parent/legal guardian to participate in the study.

## Results

### Amblyopia patients

A total of 27 anisometropic amblyopia pediatric patients (54 eyes, 59.3% girls) were enrolled in this study. Their mean age was 5.59 ± 1.08 (4–7) years old (Table 1). BCVA (logMAR) of the amblyopic eyes ranged from 0.70 to 0.22 and was significantly lower than the fellow eyes (t = 11.15, *p* < 0.001).

**Table 1:** The basic information and the refractive errors of the participants.

Serial number	Age (year)	Sex	Amblyopic eye				Fellow eye			
			S	C	A	BCVA	S	C	A	BCVA
1	7	F	2.00	− 2.75	10	0.30	0.50	− 1.50	175	0.00
2	5	F	4.00	− 1.25	25	0.30	0.00	0.00	0	0.00
3	4	F	3.50	0.00	0	0.30	2.50	0.00	0	0.05
4	6	F	2.50	− 0.50	135	0.40	0.00	− 0.50	170	0.00
5	9	F	1.50	− 1.75	180	0.22	0.50	0.00	0	0.00
6	5	M	3.00	− 2.25	175	0.30	0.00	− 0.50	25	0.00
7	5	F	− 1.00	− 2.00	25	0.40	0.00	− 1.25	150	0.05
8	6	F	1.50	− 2.25	10	0.22	0.00	− 0.50	170	0.00
9	6	F	4.75	0.00	0	0.70	1.50	0.00	0	0.10
10	4	F	5.50	0.00	0	0.22	1.00	0.00	0	0.00
11	5	M	6.50	− 0.50	160	0.30	1.25	− 1.00	5	0.05
12	7	M	7.00	− 0.75	10	0.22	0.00	0.00	0	0.00
13	4	F	2.00	− 2.75	180	0.22	0.00	0.00	0	0.00
14	5	F	2.75	0.00	0	0.22	1.00	0.00	0	0.00
15	5	F	− 2.75	− 2.00	5	0.40	0.00	0.50	5	0.05
16	6	M	2.50	− 3.50	5	0.30	0.75	0.50	5	0.05
17	5	M	7.50	0.00	0	0.30	0.00	− 1.50	180	0.10
18	7	F	8.75	0.00	0	0.52	2.00	0.50	165	0.10
19	5	M	2.50	0.00	0	0.40	− 0.75	0.00	0	0.00
20	6	M	0.00	− 2.25	5	0.40	1.00	1.50	10	0.10
21	6	M	6.75	0.00	0	0.52	2.50	0.00	0	0.00
22	5	M	2.50	− 2.75	180	0.70	0.50	0.00	0	0.00
23	6	M	0.50	− 3.00	15	0.30	2.00	− 0.75	165	0.10
24	5	M	3.75	− 1.00	5	0.70	1.00	0.00	0	0.05
25	6	F	3.50	− 1.00	15	0.52	1.50	− 1.50	180	0.10
26	5	F	2.50	− 2.25	175	0.40	0.00	− 1.50	0	0.05
27	6	F	0.00	− 1.75	155	0.22	0.00	− 0.50	15	0.00

*F* female, *M* male, *S* spherical degree, *C* cylindrical degree, *A* axis, *BCVA* best corrected visual acuity (LogMAR).

### Angio-OCT indices

**Figure 1:**
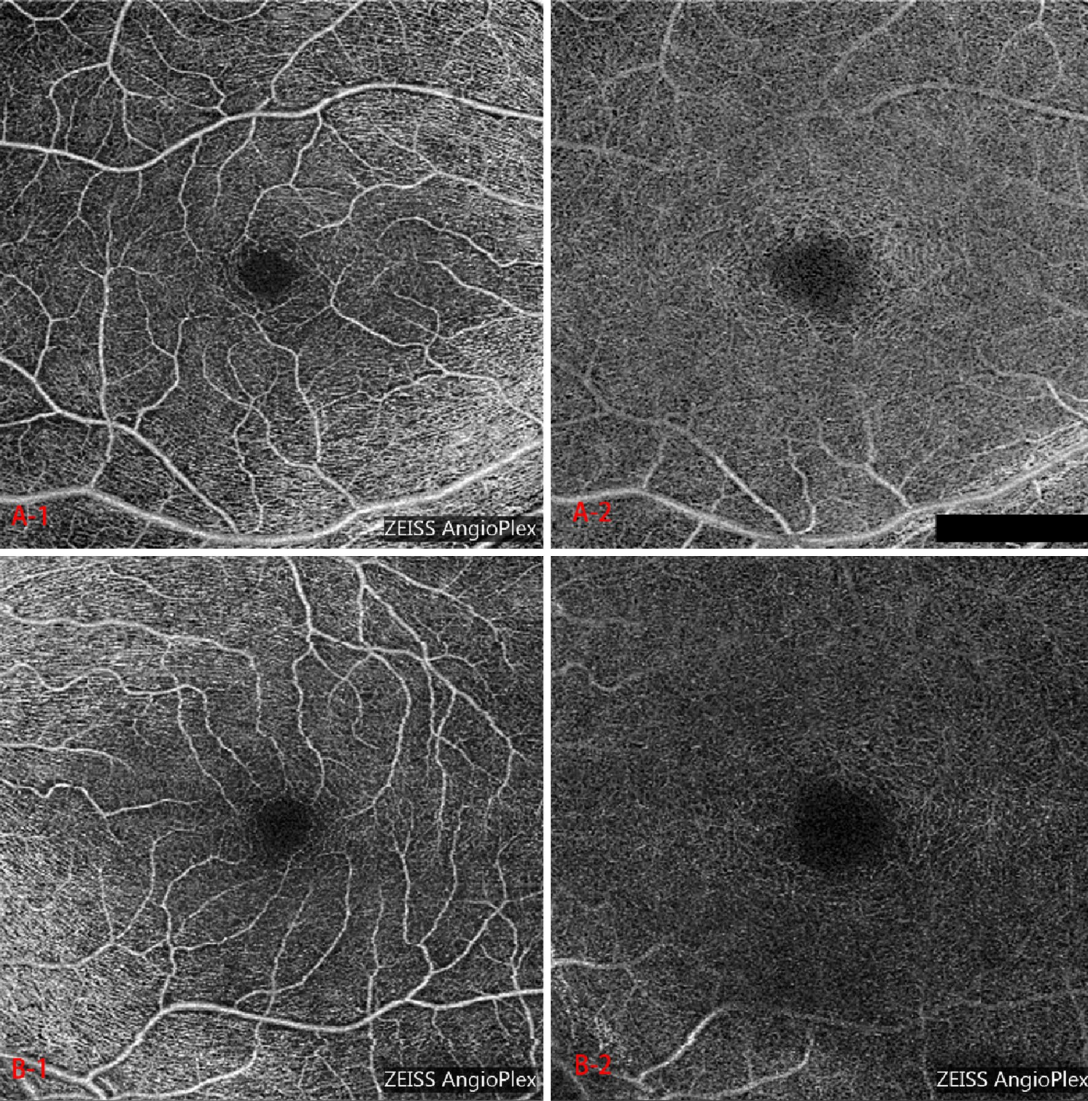
Angiography optical coherence tomography (Angio-OCT) images in superficial capillary plexus layer (SCPL) and deep capillary plexus layer (DCPL) between the amblyopic eye and the contralateral eye in one unilateral anisometropic amblyopic child. (**A-1**) and (**A-2**) showed the SCPL and DCPL respectively in the right eye which BCVA was normal. The same participant, (**B-1**) and (**B-2**) showed the SCPL and DCPL respectively in the left eye which was the amblyopic eye. The fovea avascular zone (FAZ) size of the amblyopic eye (**B-1**,**B-2**) was larger both in SCPL/DCPL.

Compared with the fellow eyes (which BCVA were normal), Angio-OCT indices (VD, VAD, VSD, IVP) at the level of the SCPL/DCPL of the parafoveal and peripapillary areas were at low value in the amblyopic eyes (*p* > 0.05). However, there was no statistically significant difference in the indices between the amblyopic and the contralateral eyes (*p* > 0.05) (Table 2). Besides, no obvious decrease in the parafoveal and peripapillary regions was observed in the four quadrants analyses of the amblyopic eyes (*p* > 0.05) (Table 3). Meanwhile, the FAZ was enlarged in SCPL/DCPL of the amblyopic eyes (0.28 ± 0.12 vs. 0.26 ± 0.11 in SCPL and 0.68 ± 0.20 vs. 0.65 ± 0.18 in DCPL) (Table 2, Fig. 1). Except for the measurement at 2000 µm and 1500 µm from the fovea at the temple (T2000 and T1500), choroidal thickness in amblyopic eyes were significantly thicken than the fellow eye (t_T1000_ = 2.64, t_T500_ = 3.10, t_subfoveal CT_ = 3.13, t_N500_ = 2.54, t_N1000_ = 2.55, t_N1500_ = 2.47, t_N2000_ = 2.29, respectively, *p* < 0.05) (Table 4).

**Table 2:** The parafoveal and peripapillary vascular parameters on SCPL/DCPL and the FAZ size in Angio-OCT.

	Amblyopia	Emmetropia	Mean	SD	t	*p*	Amblyopia	Emmetropia	Mean	SD	t	*p*
*SCPL*	*Parafovea*						*Peripapillary*					
VD	21.04 ± 0.32	21.09 ± 0.33	− 0.054	0.413	− 0.675	0.506	21.13 ± 0.31	21.21 ± 0.27	− 0.073	0.364	− 1.046	0.305
VAD	0.54 ± 0.01	0.54 ± 0.01	− 0.004	0.011	− 1.954	0.062	0.57 ± 0.02	0.57 ± 0.11	− 0.002	0.025	− 0.498	0.623
VSD	0.15 ± 0.00	0.15 ± 0.00	0.000	0.002	− 1.000	0.327	0.16 ± 0.01	0.16 ± 0.00	− 0.001	0.007	− 0.877	0.388
IVP	0.35 ± 0.01	0.35 ± 0.01	0.000	0.005	0.000	− 1.000	0.35 ± 0.00	0.36 ± 0.00	− 0.002	0.011	− 1.190	0.245
FAZ	0.28 ± 0.12	0.26 ± 0.11	0.018	0.088	1.054	0.302						
*DCPL*	*Parafovea*						*Peripapillary*					
VD	19.83 ± 0.34	19.87 ± 0.30	− 0.038	0.309	− 0.641	0.527	19.79 ± 0.42	19.90 ± 0.41	− 0.102	0.483	− 1.096	0.283
VAD	0.52 ± 0.01	0.52 ± 0.01	− 0.001	0.012	− 0.625	0.537	0.51 ± 0.02	0.51 ± 0.01	− 0.002	0.025	− 0.445	0.660
VSD	0.15 ± 0.00	0.15 ± 0.00	0.000	0.005	− 0.372	0.713	0.15 ± 0.01	0.15 ± 0.00	− 0.001	0.007	− 0.964	0.344
IVP	0.36 ± 0.00	0.36 ± 0.00	− 0.001	0.004	− 1.000	0.327	0.35 ± 0.01	0.35 ± 0.00	− 0.003	0.014	− 0.978	0.337
FAZ	0.68 ± 0.20	0.65 ± 0.18	0.030	0.161	0.981	0.336						

Without statistically significant different, Angio-OCT indices in the SCPL/DCPL were lower in the amblyopic eyes. The mean FAZ size of the amblyopic eyes was larger in SCPL/DCPL.

*SCPL/DCPL* superficial/deep capillary plexus layer, *FAZ* foveal avascular zone, *Angio-OCT* optical coherence tomography angiography, *SD* standard deviation, *VD* vessel diameter (µm), *VAD* vascular area density, *VSD* vascular skeleton density; IVP: index of vessel perimeter.

Significant statistical difference, *p* < 0.05.

**Table 3:** The parafoveal/peripapillary quadrant regions in amblyopia and emmetropia eyes.

	Amblyopia	Emmetropia	Mean	SD	t	*p*	Amblyopia	Emmetropia	Mean	SD	t	*p*
	*Each quadrant of parafoveal region in SCPL*						*Each quadrant of parafoveal region in DCPL*					
VD T	21.06 ± 0.51	21.09 ± 0.79	− 0.030	1.030	− 0.159	0.875	20.39 ± 0.50	20.24 ± 0.53	0.153	0.673	1.179	0.249
VD S	21.047 ± 0.73	21.20 ± 0.70	− 0.150	0.940	− 0.842	0.408	20.13 ± 0.69	20.26 ± 0.51	− 1.341	0.634	− 1.099	0.282
VD N	20.93 ± 0.72	20.92 ± 0.83	0.020	1.170	0.074	0.942	20.23 ± 0.59	20.27 ± 0.61	− 0.043	0.814	− 0.274	0.786
VD I	21.02 ± 0.61	20.99 ± 0.63	0.035	0.829	0.221	0.827	19.96 ± 0.63	20.04 ± 0.60	− 0.072	0.762	− 0.493	0.626
VAD T	0.53 ± 0.02	0.53 ± 0.02	0.000	0.029	0.000	1.000	0.52 ± 0.02	0.52 ± 0.02	0.003	0.024	0.651	0.521
VAD S	0.54 ± 0.02	0.54 ± 0.02	− 0.003	0.026	− 0.516	0.610	0.52 ± 0.02	0.53 ± 0.02	− 0.007	0.230	− 1.168	0.106
VAD N	0.47 ± 0.17	0.53 ± 0.03	− 0.058	0.162	− 1.871	0.073	0.53 ± 0.02	0.53 ± 0.03	− 0.003	0.030	− 0.572	0.572
VAD I	0.53 ± 0.02	0.53 ± 0.02	− 0.003	0.003	− 0.531	0.600	0.52 ± 0.02	0.52 ± 0.03	0.003	0.031	0.428	0.672
VSD T	0.15 ± 0.01	0.15 ± 0.00	0.001	0.007	0.570	0.574	0.15 ± 0.01	0.15 ± 0.01	0.000	0.006	0.296	0.769
VSD S	0.15 ± 0.00	0.15 ± 0.00	0.000	0.005	0.372	0.713	0.16 ± 0.01	0.16 ± 0.01	− 0.001	0.007	− 1.072	0.294
VSD N	0.15 ± 0.01	0.15 ± 0.01	0.000	0.007	0.000	1.000	0.15 ± 0.01	0.15 ± 0.01	− 0.001	0.007	− 0.827	0.416
VSD I	0.15 ± 0.01	0.15 ± 0.01	− 0.002	0.006	− 1.546	0.134	0.16 ± 0.01	0.15 ± 0.01	0.001	0.008	0.721	0.477
IVP T	0.36 ± 0.01	0.36 ± 0.01	0.001	0.009	0.848	0.404	0.36 ± 0.01	0.36 ± 0.01	0.000	0.008	− 0.254	0.802
IVP S	0.36 ± 0.01	0.36 ± 0.01	− 0.003	0.010	− 1.803	0.083	0.37 ± 0.01	0.37 ± 0.01	− 0.001	0.008	− 0.941	0.355
IVP N	0.36 ± 0.01	0.36 ± 0.01	0.001	0.009	0.848	0.404	0.36 ± 0.01	0.37 ± 0.01	− 0.001	0.010	− 0.386	0.703
IVP I	0.36 ± 0.01	0.36 ± 0.01	− 0.001	0.009	− 0.891	0.381	0.37 ± 0.01	0.36 ± 0.01	0.003	0.010	1.317	0.199
	*Each quadrant of peripapillary region in SCPL*						*Each quadrant of peripapillary region in DCPL*					
VD T	21.75 ± 0.85	21.65 ± 0.61	0.100	0.924	0.560	0.580	20.76 ± 0.74	20.74 ± 0.80	0.022	0.986	0.115	0.909
VD S	21.08 ± 0.42	21.02 ± 0.39	0.061	0.663	0.476	0.638	20.08 ± 0.75	19.84 ± 0.81	0.240	1.080	1.157	0.258
VD N	21.32 ± 0.49	21.39 ± 0.53	− 0.077	0.705	− 0.568	0.575	20.30 ± 0.74	20.18 ± 0.57	0.125	0.980	0.662	0.514
VD I	21.21 ± 0.65	21.17 ± 0.37	0.041	0.785	0.272	0.788	20.30 ± 1.44	20.24 ± 0.62	0.060	1.527	0.204	0.840
VAD T	0.59 ± 0.04	0.59 ± 0.02	0.000	0.038	0.000	1.000	0.55 ± 0.28	0.55 ± 0.20	0.002	0.030	0.318	0.753
VAD S	0.64 ± 0.03	0.64 ± 0.22	0.001	0.028	0.139	0.891	0.55 ± 0.03	0.54 ± 0.05	0.010	0.056	0.964	0.344
VAD N	0.61 ± 0.03	0.60 ± 0.02	0.002	0.034	0.337	0.739	0.53 ± 0.03	0.53 ± 0.21	0.001	0.034	0.171	0.866
VAD I	0.62 ± 0.04	0.63 ± 0.02	− 0.005	0.043	− 0.625	0.537	0.54 ± 0.04	0.54 ± 0.03	− 0.010	0.034	− 1.481	0.151
VSD T	0.16 ± 0.01	0.16 ± 0.01	0.001	0.011	0.348	0.731	0.16 ± 0.01	0.16 ± 0.00	0.001	0.008	0.493	0.626
VSD S	0.18 ± 0.01	0.18 ± 0.01	0.001	0.006	1.000	0.327	0.16 ± 0.01	0.16 ± 0.01	0.001	0.011	0.337	0.739
VSD N	0.17 ± 0.01	0.17 ± 0.01	0.001	0.010	0.402	0.691	0.16 ± 0.01	0.16 ± 0.01	0.000	0.009	− 0.205	0.839
VSD I	0.17 ± 0.01	0.17 ± 0.01	− 0.003	0.013	− 1.302	0.204	0.16 ± 0.01	0.16 ± 0.01	− 0.002	0.008	− 1.224	0.232
IVP T	0.36 ± 0.01	0.36 ± 0.01	− 0.003	0.014	− 1.093	0.285	0.36 ± 0.01	0.36 ± 0.01	0.000	0.014	− 0.137	0.892
IVP S	0.38 ± 0.01	0.38 ± 0.01	− 0.002	0.009	− 1.044	0.306	0.37 ± 0.01	0.36 ± 0.02	0.002	0.020	0.490	0.628
IVP N	0.36 ± 0.01	0.36 ± 0.01	− 0.001	0.012	− 0.311	0.758	0.36 ± 0.01	0.36 ± 0.01	0.000	0.017	0.000	1.000
IVP I	0.37 ± 0.02	0.37 ± 0.01	− 0.001	0.014	− 0.267	0.791	0.36 ± 0.02	0.36 ± 0.01	− 0.004	0.014	− 1.462	0.156

*SCPL/DCPL* superficial/deep capillary plexus layer, *FAZ* foveal avascular zone, *SD* standard deviation, *VD* vessel diameter (μm), *VAD* vascular area density, *VSD* vascular skeleton density, *IVP* index of vessel perimeter, *T* temporal, *S* superior, *N* nasal, *I* inferior.

**Table 4:** The CT measurement in monocular anisometropic amblyopic eyes and the fellow eyes from T2000 to N2000 at 500 µm intervals with EDI in Angio-OCT.

	Amblyopia	Emmetropia	t	*p*
T2000	354 ± 68.57	334 ± 69.04	1.47	0.155
T1500	367 ± 66.72	339 ± 65.80	2.05	0.050
T1000	383 ± 67.87	348 ± 71.76	2.64	0.014*
T500	396 ± 70.78	351 ± 76.82	3.10	0.005*
Subfoveal CT	409 ± 78.00	358 ± 84.23	3.13	0.004*
N500	381 ± 77.33	340 ± 85.76	2.54	0.017*
N1000	359 ± 75.00	316 ± 90.89	2.55	0.017*
N1500	338 ± 85.02	294 ± 90.64	2.47	0.020*
N2000	306 ± 95.03	263 ± 87.55	2.29	0.030*

*CT* choroidal thickness, *T2000* from the fovea to the temple 2000 µm, *N2000* from the fovea to the nasal 2000 µm, *EDI* enhanced depth imaging, *Angio-OCT* optical coherence tomography angiography.

*Significant statistical difference, *p* < 0.05.

## Discussion

This present study compared the choroidal and retinal microstructural vascularity between the amblyopic and the fellow eyes in anisometropic amblyopia. The observations from this present study suggests that anisometropic amblyopia does not only affect the visual cortex but also affect the choroidal thickness. In particular, there was significant difference in the subfoveal area and all the parafoveal areas except for the measurements taken from 2000 and 1500 µm temporally. As we all know, the choroid provided nutrients to the retinal outer part and blood supply to the optic nerve, which accounted for the majority of the ocular blood flow. The layers of choriocapillary maintained and provided the outer retina as the primary blood supply of for the photoreceptors. In response to adjust the retinal position and promote visual development, the choroid thicknesses became thinner. Studies had shown the choroid played a role in emmetropization and refractive error development^21,22^. However, this choroidal compensation did not occur in amblyopic eyes^14^. Recently, by a study of 14 amblyopes aged 6–12 years, Kaur et al. found that the VD in choriocapillary attenuated in the amblyopic eye which suggested that the choriocapillary may involve in the amblyopia pathogenesis^23^. Many researchers had confirmed that the CT in amblyopic eyes was thicker than in the fellow eyes and the normal controls, which was consistent with our results^14,16,24,25^. But, different results about changes in choroid had been observed by Kurt et al. and colleagues. They found the CT in the amblyopic and non-ambliyopic fellow eyes did not show statistical significance and suggested that amblyopic eyes have the same high metabolic activity as the fellow eye in the same patient^26^. These different conclusions may be attributed to sex, axial length, age, and variation of choroidal diurnal variation^26–28^.

Contrast to the choroid, researchers found that amblyopia did not affect retinal thickness^29^. Histologically, SCPL, intermediate capillary plexus layer (ICPL), DCPL and radial peripapillary capillaries layer (RPCL) composed the retinal vascular network. In the macular region, both at the levels of SCPL and DCPL sharply demarcated a ring of FAZ. In the peripapillary region, RPCL supplied supply the retinal nerve fiber layer (RNFL). Fundamental neurological studies showed that the amblyopia development was associated with the variation in retinal microcirculation and function, and these were supported by clinical research^30^. The microvessel changes in the parafoveal/peripapillary areas may represent the variation that the amblyopic eyes suffered. In accordance with many researchers, we found the Angio-OCT indices of the SCPL/DCPL in the parafoveal areas were decreased in amblyopic eyes compared with fellow emmetropia eyes^31–33^. Yilmaz and colleagues hypothesized that the underuse of the amblyopic eyes may induce these retinal or choroid microvasculature alterations^32^.

The FAZ, which was sharply surrounded by a capillaries ring demarcating the avascular area, presented both at the levels of SCPL and DCPL in the macular region. Similar with Yilmaz’s research, we found that the FAZ in the anisometropia amblyopic eyes at the level of SCPL/DCPL were larger than the fellow eyes, though without statistically significant difference. However, Isa Sobral et al. documented a marginally statistically significant increase in FAZ of DCPL in amblyopic eyes compared with control eyes^33^. Differently, Araki and partners’ research showed no significant difference in the macular VD but smaller FAZ area of SCPL was found in the amblyopic eye after magnification error correction^9^. We analyzed that the inconsistence may due to the amblyopic classification and degree, as many previous reports enrolled both the anisometropia and the strabismus amblyopic patients or only with the strabismus amblyopic children. Besides, the small sample and the age of the participants also may lead to the inconsistent. Furthermore, the difference of built-in software algorithm in Angio-OCT should be considered.

Of note, though without significant difference, we observed the vascular perfusion in the parafoveal region/peripapillary region and found that the vascular indices were decreased in the amblyopic eyes both on the SCPL and DCPL level. Besides, no obvious decrease in the parafoveal and peripapillary regions was observed in four quadrants analyses of the amblyopic eyes (*p* > 0.05). Until now, we have a small age span range (4–7 years old) and we aim to minimize the age influence on our results. The vessel density parameters reflected the vessel dimension and vascular supply changes at different regions in the fundus. Previous reports showed discrepancy of opinions on the microvascular structure and function. Sobral et al. pointed that not only the microvascular structure changed, but also function dependent parameters were influenced in the macula and the optic nerve among amblyopic children^33^. Unlikely, Cinar E hold the viewpoint that no significant vascular damage was demonstrated by Angio-OCT in amblyopic eyes, which was similar with our results. Although without the statistical difference in our results, we supposed that minor vascular perfusion affected by amblyopia may correlate with macular and the optic disk blood supply. However, how these changes occur and its specific mechanisms need to be evaluated in future study. Yet, Cinar E indicated specific localized vascular defects were related to amblyopia and pointed that parameters in superior quadrant were lower in amblyopic eyes, which were inconsistent with our findings^34^. We speculated that the inconsistencies between our analysis and the literature may be related to the age distribution of the patients as well as the duration and severity of amblyopia.

There still were some limitations in our present study. Firstly, we did not categorize subjects by amblyopia severity degree. After the Angio-OCT examinations and the diagnosis of amblyopia, all the amblyopic children underwent appropriate optical correction, occlusion/penalization for the non-amblyopic eye or amblyopia training therapy. However, we did not document the amblyopic children’s BCVA recovery or analyze their microstructural vascularity after treatment. Moreover, in order to eliminate the differences caused by different individuals, we chose the paired comparison between the amblyopic and the fellow eyes in anisometropic amblyopia enrollments. Indeed, though with normal BCVA, the contralateral eye in the unilateral amblyopia child may not really normal in functional sense, and we will supplement the comparison study of the amblyopic eye and the contralateral eye in the future study. Thirdly, Sobral et al. hypothesized that the brain can compensate for the vascular amblyopic deficit when amblyopia was cured^33^. We did not involve the relationship between the brain and the retinal/choroidal vessels, and we will do more observation on this in future research. Moreover, due to the differences in Angio-OCT models and images analysis algorithm, there was no reference of large sample normal groups in children for comparison. In this sense, the establishment of pediatric Angio-OCT data in normal children is crucial to determine.

## Conclusion

Compared with the contralateral eyes, the CT in certain areas were thicker in the amplyopic eyes. Besides, the retinal and optic disk vascular density indices in the SCPL/DCPL were lower in amblyopia eyes though without statistically difference. Angio-OCT may be an effective method to supervise and assess the variation of retinal and choroidal microvascular system in amblyopia pediatric patients.

## Data Availability

Some or all data, models, or code generated or used during the study are available from the corresponding author by request.
